# Group flow: A scoping review of definitions, theoretical approaches, measures and findings

**DOI:** 10.1371/journal.pone.0210117

**Published:** 2018-12-31

**Authors:** Fabian Pels, Jens Kleinert, Florian Mennigen

**Affiliations:** Department of Health & Social Psychology, Institute of Psychology, German Sport University, Cologne, North Rhine Westfalia, Germany; Università Cattolica del Sacro Cuore, ITALY

## Abstract

The purpose of this article is to provide a scoping review of the current literature on group flow. Based on the PRISMA-guidelines for systematic reviews, 26 publications were identified that met the inclusion criteria. Publication analyses comprised an individual consideration of each publication and a systematic, integrative synthesis of all publications. Analyses identified heterogeneous group flow definitions across publications, supporting the need for an integrative definition. Further heterogeneity existed in the theoretical approaches and measures used, highlighting the need for a comprehensive theory and a measurement standard. Components (e.g., synchronization), antecedents (e.g., trust), and outcomes (e.g., well-being) of group flow were identified in publications that presented empirical studies, some of which that showed similarities between characteristics of group flow and individual flow and others that showed aspects unique to group flow. Overall, this scoping review reveals the need for a systematic research program on group flow.

## Introduction

Many people enjoy the state of total immersion in a task accompanied by an optimal level of functioning, be it at work (e.g., [1]), in sports (for an overview, see [2]), in music (for an overview, see [3]), during leisure time (e.g., [4]) or potentially in any other imaginable context of our everyday life [5]. This state is referred to as flow. Flow is associated with a variety of positive outcomes, such as positive affect and enhanced performance (for an overview, see [6]). In the past decades, a multitude of studies has examined and established a concept of flow in individuals (which we refer to as individual flow) based on the initial work of Csikszentmihalyi [7]. Additionally, in recent years, there has been growing interest in flow that emerges during group situations. This kind of flow, henceforth referred to as group flow, has often been described in metaphorical terms [8], such as a state in which “things [in a group] are *clicking* or in *sync*” (p. 157) and wherein “everything [in the group] seems to come naturally” (p. 158) for example. However, as stated earlier by Nakamura and Csikszentmihalyi [9], there is not yet a clear and consistent concept for group flow. Therefore, the overall purpose of the present paper is to systematically review the recent literature on group flow in order to pave the way for a more common understanding and examination of group flow in the future.

The starting point of the development of a group flow concept was Csikszentmihalyi’s [7] concept of individual flow, describing individual flow as a rewarding experience of pure enjoyment and absorption in a task. The framework of the individual flow concept consists of nine dimensions that are typically said to make up individual flow [10]: (1) an above-average balance between the challenges of and the related individual skills for a given task, (2) clear goals for the task and (3) unambiguous, ongoing feedback on the progress of task accomplishment, (4) concentration on the task at hand, (5) a merging of action and awareness, (6) loss of self-consciousness, (7) a sense of control, (8) a transformation of time, and (9) an autotelic experience. Some researchers classify these dimensions into proximal conditions (1–3) of individual flow and the characteristics of the subjective state (4–9) while being in individual flow [9, 11].

Studies show that the experience of individual flow is not restricted to solitary behavior and that it can also occur in social situations (for an overview, see [12, 13]). Social situations range from situations in which persons attend to individual tasks with others merely being present (e.g., co-participants who also accomplish an individual task) to situations with highly interdependent tasks wherein others form an integral part of task accomplishment (e.g., cooperation in a group; [14]). In terms of individual tasks with others being present, a study from the recreational physical activity domain found individual flow to occur slightly more often with a co-participant than alone [15]. In terms of interdependent tasks, several studies have found that people experience individual flow in group settings, for instance, when playing in an interdependent music ensemble (e.g., [16]) or during interdependent sports team (e.g., football, rowing; [17, 18]). During such tasks, an individual may be in an individual flow state regardless of whether the individual’s co-participants or interdependent group members are in an individual flow state or not [9].

Besides these individual experiences, recent literature (e.g., [9, 14, 19]) assumes that a specific kind of flow can occur during group situations (i.e. group flow) that qualitatively differs from individual flow. This assumption is based on two sources. First, this assumption is based on anecdotal, non-peer-reviewed evidence for a phenomenon of group flow gained from creative groups. For example, while studying individual flow, Sato [20] found that acting in creative motorcycle gangs evoked “a shared experience of collective effervescence” (p. 116). Collective effervescence, a sociological concept developed by Durkheim in the beginning of the 20^th^ century, describes the excitement of participating in communal gatherings [21]. Second, the assumption is based on the results of noteworthy classic research from the field of social psychology. This research, as outlined by Walker [14], found that group contexts introduce many additional variables that cause individuals to act, think, and feel differently during group situations compared to solitary situations. Consequently, these variables and differences “may inhibit, facilitate, or transform flow experiences” ([14], p. 4). These issues suggest that group flow might exceed the simple experience of individual flow in a group setting.

In contrast to individual flow, group flow may comprise a specific experience of (being in a) group and an experience of interpersonal action as it takes place in a group situation. For these experiences to occur, there is the need for a group situation in which a group is (a) psychologically *and* physically present (i.e. it is not sufficient to have a group only psychologically present, as in the minimal-group-paradigm; e.g., [22]) and in which there is (b) an explicit group task (e.g., completing an interactive group task) or an implicit group task (e.g., everyone is doing a task on his/her own, but each individual is aware that the others are doing the same). A group situation such as this would inevitably establish an experience of group that may be specific under group flow.

As a result of the points raised above, a number of authors began examining the phenomenon of group flow. In the course of these examinations, the need to identify emergent qualities of group flow (e.g., [9]), conditions of group flow (e.g., [14]), or consequences of group flow (e.g., [23]) has been highlighted. However, the related literature describes solely heterogeneous approaches and, thus, homogeneous conceptualizations of group flow are missing.

Therefore, this scoping review addresses four specific aims: firstly, to present an overview of recent definitions of group flow; secondly, to provide an overview of recent theoretical approaches to group flow; thirdly, to review previous measures assessing group flow; and finally, to review empirical findings on group flow. The achievement of these aims is associated with two potential benefits, the occurrence and usability of which depends on the quality of the results gained from previous literature: firstly, amalgamating the range of existing literature can pave the way for future research on group flow; secondly, this work can support practical applications of a group flow concept (e.g., helping to improve the practical realization of group flow experiences in the field), for instance, by superiors (e.g., leaders of a work group, the maestro of an orchestra, or the coach of a football team) seeking to make use of group flow in order to attain potentially positive outcomes for their respective group and group members.

## Method

### Eligibility criteria

The scoping review was carried out based on the PRISMA guidelines [24, 25] for systematic reviews (without registering a protocol). Eligibility criteria for identified articles included: (1) peer-reviewed publication, (2) publication in English, and (3) publication explicitly dealing with group flow. Studies examining individual flow in solely a social situation were excluded. There were no restrictions made regarding the publication date or methodological approaches (e.g., study design, sample size, context of investigation) of empirical studies.

### Search strategy and information sources

The literature search process was twofold. Primarily, the literature search was undertaken using the psychology databases PsycINFO, PsycARTICLES, and PSYNDEX. Different search terms for group flow and potential synonyms were applied at the same time and entered into the search box TX (“All Text”) within parentheses: ("*team flow" OR "flow in a team" OR "flow in team*" OR "team* in a flow" OR "team* in flow*" OR "*group flow" OR "flow in a group" OR "flow in group*" OR "group* in a flow" OR "group* in flow" OR "interpersonal flow" OR "social flow" OR "collective flow" OR “shared flow”). In order to avoid findings on blood flow and animal studies, these search terms were added with the formula NOT (blood OR animal), which was entered into the search box TI (“Title”) in parentheses. A limiter was applied to restrict results to peer-reviewed articles. The final database search was conducted in June 2018.

Secondarily, the literature search included the consultation of reference lists of retrieved articles, book chapters, and publication lists of authors who regularly publish in this area (accessed via their websites) to locate additional references of interest.

### Publication selection

Duplicate publications were automatically removed by the provider of the databases during database searches (primary literature search). During additional manual record searches (secondary literature search), duplicates were excluded from the beginning. Afterwards, the title and abstract of each publication were screened, followed by–if necessary–a full-text article assessment for eligibility.

### Publication analyses and data extraction

Publication analyses comprised an individual consideration of each publication and, based on this, a systematic, integrative synthesis of these publications. For all publications, individual consideration involved the extraction of (1) the group flow term used (e.g., group flow, team flow, collective flow etc.), (2) the underlying group flow definition (i.e. the precise wording of the definition), and (3) the underlying theoretical approach (i.e. the theoretical model used and its assumptions). Additionally, in case of a publication with empirical data, the (4) context of the investigation (e.g., work, education, music), (5) sample characteristics (i.e. sample size, gender, age, specific study-related characteristics; if available), (6) study design (i.e. cross-sectional, longitudinal, or experimental), (7) group flow measure, and (8) study results (with regard to group flow) were extracted. Extraction of the (7) group flow measure comprised the consideration of the approach (i.e. qualitative or quantitative), the perspective of the measure (i.e. self-report or external perspective), and whether the measure fits the group flow concept. The measures were defined as not fitting the group flow concept (i.e. to be inappropriate) when (a) the measure actually assessed individual flow or (b) the measure actually assessed individual flow and aggregated this to a group flow value per group of investigation. This is in line with the pioneering work on group flow of Sawyer [26], which states that group flow is more than just an aggregation of individual flow. Thus, results of studies with an inappropriate group flow measure were excluded from extraction of (8) study results. Extraction of study results comprised an identification of antecedents, components (i.e. characteristics), consequences, and correlates of group flow that the respective study found, irrespective of the assessment and statistical procedure used by the study. For this purpose, the underlying study design was also taken into account (e.g., if a study claimed to have identified consequences of group flow although the investigation was cross-sectional in nature, the respective construct was classified as a correlate and not as a consequence). Data were extracted by two authors.

The systematic, integrative synthesis was guided by the four aims of this review. Specifically, it involved the narrative description of (1) the definitions of group flow, (2) the theoretical approaches to group flow, (3) the measures of group flow, and (4) the empirical findings on group flow. The integrative narrative description of (4) empirical findings was done with respect to the underlying context of investigation and study design. Thus, the presentation of empirical findings is always accompanied by information about the underlying context and the study design in order to contextualize the findings. The methodological qualities of the studies were also taken into account during the final interpretation of the results in the discussion section.

## Results

### Publication selection

**Fig 1:**
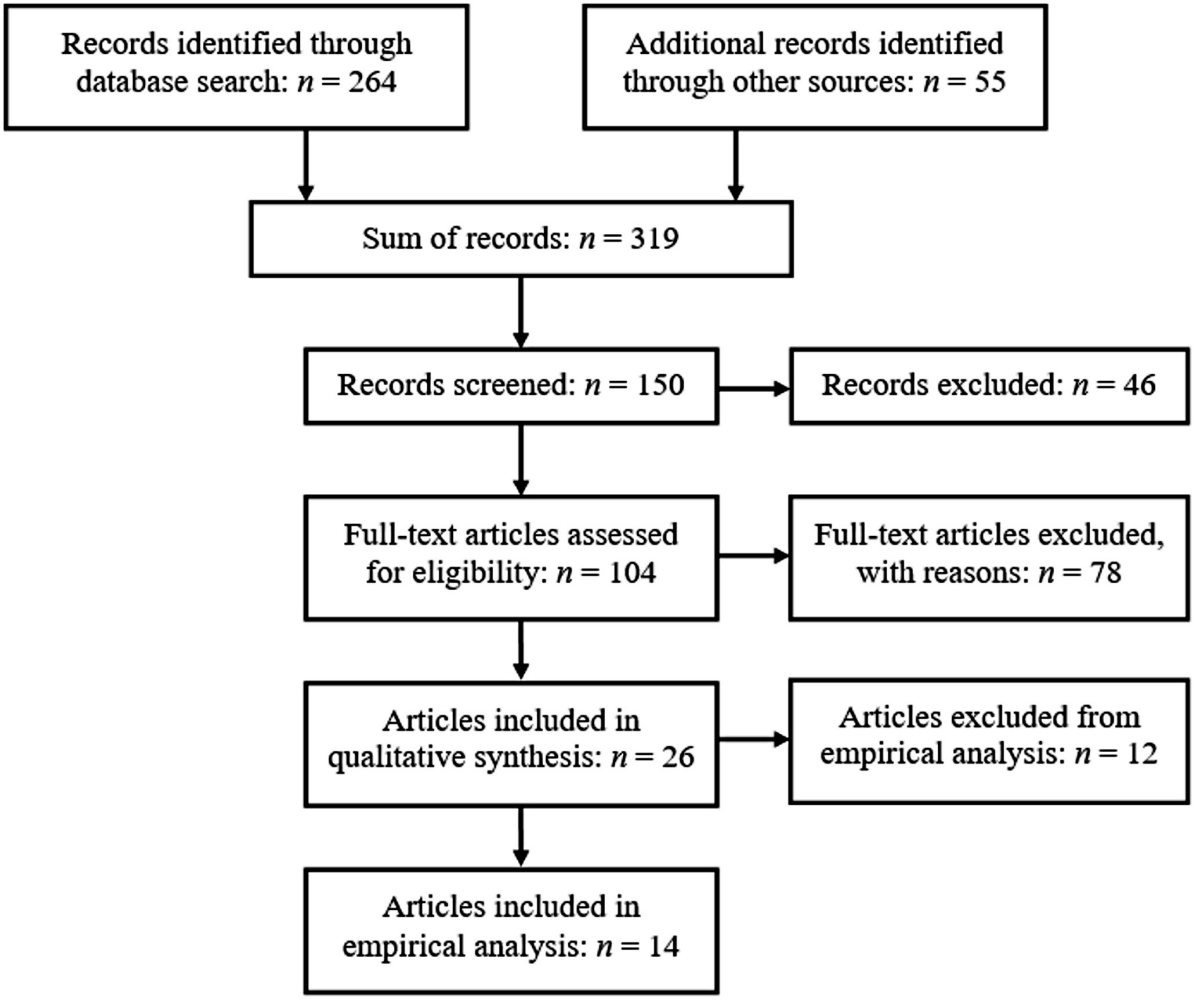
Flow chart of reviewed studies. Duplicates were removed automatically by the provider of the databases during database search; during additional manual record search (other sources), duplicates were excluded from the beginning.

Through the primary search (databases), 264 publications were identified (Fig 1). A further 55 publications were identified through the secondary search (consultation of reference lists of retrieved articles, book chapters, and publication lists of authors who regularly publish in this area). The title and abstract of these 319 publications were subsequently screened, leaving 104 publications to be assessed for eligibility. For various reasons, 78 publications did not meet the inclusion criteria (e.g., publication on individual flow in a social setting, publications on flow of group communication, publications on blood flow). Finally, 26 publications were included in this review. Of these, 22 were original empirical articles and four were non-systematic literature review articles. Twelve studies were excluded from the analysis of empirical findings because of inappropriate group flow measures: For instance, Keeler and colleagues [27] describe group flow as involving optimal interaction with others, but their measurement tool, the Flow State Scale-2 [28], assesses individual flow experience only and cannot be said to capture optimal interaction between group members (see Method section). In other words, although all of the included studies conceptualized group flow as different from individual flow, some made use of typical individual flow measures to measure group flow. Consequently, only the results of the studies that actually assessed (a kind of) group flow and did not measure individual flow were further analyzed.

### Definitions of group flow

Table 1 displays the construct designations and the definitions of group flow in past publications. In terms of construct designations, there were thirteen different terms used to label group flow (collective flow, combined flow, contagious flow, flow in teams, group flow, networked flow, shared flow, social flow, team flow, team-level flow, flow state on teams, flow in groups, team flow state). Of these, group flow was the most frequently used term (*n* = 13). Some authors used more than one of these terms interchangeably within the same publication (e.g., [29, 30]).

**Table 1:** Group flow designations, definitions and theoretical approaches of reviewed studies.

Reference	Designation	Definition	Individual aspects of group flow	Collective aspects of group flow	Theoretical Approach
Admiraal et al. [31]	Team flow				
Armstrong [32]	Group flow	“(…) a collective state that occurs when a group is performing at the peak of its abilities (…).” (p. 102)		Group performing at the peak of its abilities	Group Flow concept [26]
Aubé et al. [33]	Flow in teams	“(…) within teams, flow can become a collective phenomenon given that members share the same work experience and that this psychological state may have a ‘contagion effect’ (…).” (p. 122)		Members share the same work experience; contagion effect	
Bakker et al. [34]	Team-level flow	“(…) team-level flow may be the result of contagion effects, where [individuals] transfer their own moods and behaviors to [others] in their team.” (p. 443)		Contagion effect (transfer of mood and behavior)	
Culbertson et al. [29]	Contagious flow collective flow/ /social flow	“(…) when in a group setting (…), the experience of flow is a social phenomenon in which the presence of others is used to gauge one’s own flow experience.” (p. 323)		Presence of others is used to gauge one’s own flow experience	Social Comparison Theory [35]; Social Validation [36]; Emotional Contagion Theory [37]
Duff et al. [38]	Group flow/networked flow	“When a team is in flow, it is innovative, harmonious and productive. Being part of it improves the performance of each member. Communication is purposeful and clear. Friction is seen as an opportunity, not a personal threat. The balance is just right, and everything flows.” (p. 575)	Improved performance of each member; friction is seen as an opportunity, not as a personal threat	Team is innovative, harmonious, productive; clear and purposeful communication; right balance	Multi-level model of flow in sociotechnical systems [38]
Gaggioli et al. [39]	Group flow/networked flow	“(…) ‘a collective state of mind (…) [sic] a peak experience, a group performing at its top level of ability’ (…).” (p. 41)	Peak experience	Collective state of mind; group performing at its highest level of ability	Networked Flow Model [39]
Gaggioli et al. [40]	Group flow/networked flow	“(…) an optimal collective experience defined as a ‘collective state of mind’ (…).” (p. 158)		Optimal collective experience; collective state of mind	Networked Flow Model [39]
Gaggioli et al. [41]	Networked flow	“(…) a ‘collective state of mind’ (…).” (p. 2)		Collective state of mind	Networked Flow Model [39]
Galimberti et al. [42]	Group flow	“When high social presence is achieved, participants can enjoy an optimal state that maximizes the creative potential of the group (Networked Flow, NF). The adjective «networked» is used to stress the conceptualization of NF as a systemic emergence, resulting from the micro-interactions between the components of the group (…).” (p. 33)	Enjoyment	High social presence; creative potential of the group; micro-interactions between the components of the group	Networked Flow Model [39]
Gloor et al. [43]	Shared flow/combined flow/group flow	“If a team is collectively in [individual] flow (what we call ‘group flow’) it therefore will deliver high performance (…). Group flow is based on flow experienced in relational embeddedness (…) which in itself increases satisfaction (…).”(p. 38)	Individual flow	Collectively in individual flow; high performance; relational embeddedness	
Hart & Di Blasi [19]	Group flow	“[Shared flow] is currently characterized by group activities such as ‘hot groups’, (defined as absorbing, vital and hard-working interactive teams or task forces (…)), and musical jam sessions (…). In these groups it is expected that all those involved are experiencing the nine characteristics of individual flow while concurrently engaging in a shared goal-oriented activity (…).” (p. 278)	Individual flow	Concurrent engagement in a shared goal-oriented activity	
Heyne et al. [30]	Flow state on teams/flow in groups/team flow state/flow in teams				
Kaye [44]	Group flow				
Kaye & Bryce [45]	Group flow/shared flow				
Kaye & Bryce [46]	Group flow	“(…) group flow is conceptualized as an experience shared between a number of individuals, which enables each of them to achieve [individual] flow as the result of common focus on parallel and organized tasks, shared social belonging, and collective competency (…).” (p. 50)	Individual flow	Experience is shared between individuals; common focus on parallel and organized tasks; collective competency; shared social belonging	
Keeler et al. [27]	Social flow	“[Social flow] involves not only optimal performance, but also optimal interaction with others (…).” (p. 3)		Optimal performance with others; Optimal interaction with others	
Kiili et al. [47]	Team flow				Extended channel model [47]
MacDonald et al. [48]	Group flow				
Primus & Sonnenburg [49]	Group flow				Group Flow concept [32]
Ryu & Parsons [23]	Social flow/collective flow				
Salanova et al. [50]	Collective flow	“[Collective flow is an experience that] happen[s] at the group level as a kind of shared positive experience.” (p. 436)		Shared positive experience	Social Cognitive Theory [51]
Sawyer [8]	Group flow	“When a group is performing at its peak, I refer to it as being in group flow, in the same way that an individual performing at his or her peak often experiences a subjective feeling of flow. (…) group flow is a property of the entire group as a collective unit. (…) In group flow, everything seems to come naturally; the performers are in interactional synchrony (…). In this state, each of the group members can even feel as if they are able to anticipate what their fellow performers will do before they do it.” (p. 158)		Group performing at its peak; interactional synchrony; everything seems to come naturally; group members feel as if they are able to anticipate what their fellow performers will do before they do it	Group Flow concept [26]
Walker [14]	Social flow	“Social flow should be similar to solitary flow because the basic conditions for flow for individuals must be met first, namely, emergent challenges from the environment must be matched with the skills of individuals who are freely doing meaningful tasks. However, more than a social context may distinguish social flow from solitary flow. Flow in a social context may be a qualitatively different phenomenon than flow experienced in isolation.” (p. 3f.)	Similar to solitary flow; challenges from environment matched with skills; freely doing meaningful tasks	Unspecific	
Zumeta et al. [52]	Shared flow	“During optimal experiences, particularly collective ones [like shared flow], participants transcend their ego, get involved in a more complex action system, and the individual feels one with the group he/she acts with (…).” (p. 3)	Transcendence of ego; feeling one with the group one is acting with	Collective optimal experience; complex action system	Social Cognitive Theory [51]
Zumeta et al. [53]	Shared flow	“[Shared flow is] a state of synchronized collective optimal experience. (…) In the state of shared flow, all members of the group experience the same sensation of being absorbed by the activity.” (p. 717f.)	Absorption by the activity	Synchronized collective optimal experience	

In terms of definitions, 18 out of the 26 publications provided a–more or less–explicit definition of group flow. These definitions can be decomposed into different elements. These elements relate to (1) *individual aspects* and (2) *collective aspects* of group flow.

1. *Individual aspects* relate to the question of how an individual experiences group flow. Of the 18 definitions, nine mention individual aspects. Of these, five definitions [14, 19, 43, 46, 53] describe the typical characteristics of individual flow to also be characteristic of the individual experience of group flow (e.g., immersion in the activity). In addition, other definitions comprise individual aspects such as enjoyment [42] or feeling one with the group that one is working with [52].
2. *Collective aspects* relate to features of a group as a whole being in group flow. All of the 18 definitions mention collective aspects of group flow. These collective aspects can be differentiated into four categories: (a) a specific shared state, (b) a specific group performance, (c) a specific group interaction, and (d) a specific social constellation. The specific *shared state* comprises a collective state of mind (mentioned in the three definitions of the same author group; [39–41]) and an optimal collective experience (mentioned in seven definitions; e.g., “shared positive experience”, [50], p. 436; “synchronized collective optimal experience”, [53], p. 717). Furthermore, one definition characterizes this state as a group that is collectively in individual flow (mentioned in one definition; [43]). The specific *group performance* comprises an optimal collective performance at a high ability level (mentioned in six definitions; e.g., “group performing at its top level of ability”, [39]) and a creative potential of the respective group being in flow (mentioned in one definition; [42]). The specific *group interaction* comprises a positive interaction between the group members (mentioned in three definitions; e.g., “interactional synchrony”, [8], p. 158) and a shared task (mentioned in three definitions; e.g., “shared goal-oriented activity”, [19], p. 278). Social contagion between group members was also mentioned (two definitions; [33, 34]). Finally, the specific *social constellation* comprises the high social presence of others in a given action system (mentioned in three definitions; e.g., “high social presence”, [42], p. 33) and a positive relationship quality between an individual and the others that are present (mentioned in two definitions; e.g., “relational embeddedness”, [43], p. 38).

### Theoretical approaches to group flow

All of the publications reviewed were principally based on Csikszentmihalyi’s Flow Concept [7, 9, 10], with only one study [42] not directly, but indirectly mentioning Csikszentmihalyi. However, only twelve publications elaborated, presented, or used a specific theory of group flow in general or a well-established psychological theoretical approach to explain the occurrence of group flow (for an overview of theoretical approaches, see Table 1).

The so-called *Group Flow concept* was used by three publications [8, 32, 49]. It states (as developed by Sawyer [26]) that group flow is not just an aggregation of individual flow, but a collective phenomenon. This collective phenomenon is represented by an interactional synchrony of the group members. The interactional synchrony is an emergent property of the group (and not an individual state of consciousness) that is said to result in positive consequences (e.g., creativity). For group flow, as an emergent property, to be more likely to occur, Sawyer [26] assumes the need for a balance between the extrinsic goals and the shared and used pre-existing structures of the group members. The more extrinsic a goal is, the more pre-existing structures are required. Extrinsic goals range from being unknown (e.g., the skit of an improvisation music group) to known (e.g., evidence for a formula that needs to be produced by a group of mathematicians). Pre-existing structures are “performance-related elements that are associated with a ritualized performance” ([26], p. 168), such as an overall outline of the performance that is known by all group members or by pre-defined roles for each group-member. In addition to this, group flow is assumed to require parallel processing between group members. Parallel processing means that group members must simultaneously concentrate on and immediately respond to each other’s actions via different senses (e.g., hearing each other, seeing each other) in order to keep the interactional synchrony flowing.

The *Networked Flow Model* was used in four publications [39, 41, 42]. Building upon Sawyers [26] Group Flow concept, this model (as developed by Gaggioli and colleagues [39]) states six phases in the development of group flow or “networked flow”, as they call it. These stages are (1) meeting (persistence), (2) reducing the distance, (3) liminality-parallel action, (4) networked flow, (5) networked flow–creation of an artifact, and (6) networked flow–application of the artifact in a social network. Phases (1) to (3) include the forming of a group with (at least partly) shared intentions, similarities between group members and group identity. These factors are said to lead to the emergence of social presence and collective intentions. Phases (4) to (6) aim to describe the final state of group flow, which is especially characterized by “a state of mind characterized by a high level of concentration, involvement, control of the situation, clarity of objectives, intrinsic motivation and a positive emotional state” (p. 45). Precedents of group flow in this model are collective action based on collective intentions, future-oriented and internalized collective intentions, a balance of resources and requirements, identification of group leaders, and explicit definition of the “new frame” (i.e. the group). According to the Networked Flow Model, the result of the group flow process is an “artifact” (i.e. some sort of product) that is applied to the social context of the group in the last phase of the process.

A *multi-level model of flow in sociotechnical systems* was developed by Duff and colleagues [38]. This model extrapolates the original Flow Concept of Csikszentmihalyi’s work group [7, 9, 10] across different levels. It assumes that flow is an isomorphic construct that occurs in a similar way at three different levels: (1) On the lowest level (i.e. the individual level), typical individual flow occurs. (2) On the mid-level (i.e. the group level), flow occurs in a group as a whole (i.e. group flow). This can either be co-active flow (while working on an individual task in the company of others) or interactive flow (while collaborating on a task with others). In flow, the team processes are harmonious, coordinated and the group’s communication is purposeful and clear. These processes result in innovative products. (3) On the highest level (i.e. the system level), flow occurs in a system (i.e. the entirety of all components, including individuals, teams, immediate environment, technology etc.) as a whole. However, Duff et al. [38] do not describe characteristics of system level flow.

A *channel model of group flow* was developed by Kiili et al. [47]. This model also extrapolates the original Flow Concept of Csikszentmihalyi’s work group [7, 9, 10] by extending the traditional channel model. The traditional channel model (as one component of an early version of the Flow Concept) assumes that individual flow occurs whenever there is a balance between challenges and skills for a given task (be it low challenges and low skills, medium challenges and medium skills etc.), with the corridor of balance having the name “channel”. Kiili and colleagues [47] add a team flow dimension to the traditional channel model, assuming a wider range of the challenge-skill-balance to allow for group flow to occur (i.e. the group’s overall skills can be a bit higher or lower than the challenges) compared to a very restricted range of challenge-skill-balance for individual flow (i.e. necessity of a perfect fit between above-average challenges and skills).

Furthermore, three publications [29, 50, 53] used well-established psychological theories to explain the occurrence of group flow. In more detail, *Social Comparison Theory* [35], the principle of *social validation* [36] and *Emotional Contagion Theory* [37] were considered by Culbertson et al. [29] as theoretical underpinnings of the occurrence of group flow. Social Comparison Theory states that people evaluate themselves through comparison with others. Applied to group flow, this means that individuals compare themselves to other group members in terms of engagement and immersion in a task, which guides an individual’s engagement and immersion, and, in turn, facilitates the occurrence of group flow. Additionally, this could be facilitated by social validation (i.e. observing the behavior of others to decide how to behave adequately in a given situation; [36]). Emotional Contagion Theory assumes cross-over effects of emotions between interacting people. This can explain the spreading of flow experiences from one group member to the next.

The implications of *Social Cognitive Theory* (e.g., [51]) for group flow were considered by two publications [50, 53]. Social Cognitive Theory understands people as self-organizing, proactive, self-regulating agents, whose efficacy beliefs have an important influence on their behavior (e.g., [51]). Thus, it is not only a challenge-skill-balance that is potentially important for group flow to emerge, but also a balance between challenge and group efficacy beliefs. To wit, a group does not only need sufficient skills to master a challenge, but also needs to believe that its skills are sufficient.

### Measures of group flow

In total, 22 publications that empirically investigated group flow used a group flow measure (see Table 2). In addition, three publications that did not empirically investigate group flow proposed specific group flow measures that could be used in the future. Thus, with one exception [8], all publications used or suggested a group flow measure.

**Table 2:** Summary of empirical information on reviewed studies.

Reference	Context	Sample	Design	Group activity (name or description; group size)	GF measure (qualitative/quantitative, internal/external perspective)	Main empirical findings
Admiraal et al. [31]	Education	*N* = 216 students (age: 12–16) divided into 54 groups	Cross-sectional	Digital learning (playing the game “Frequency 1550”; 4)	Structured observations of groups (qualitative, external)	GF is positively related to group performance; GF is not related to learning
Armstrong [32]	Education	Grade 8 middle school students divided into two groups	Cross-sectional	Open-ended mathematical problem solving (solving the “The Train Problem”; 4–6)	Structured observations of groups (qualitative, external)	Decentralization and trust are antecedents of GF
Aubé et al. [33]	Education	*N* = 395 undergraduate and graduate business students (201 m, 194 f; age: *M* = 28.7, *SD* = 6.5) divided into 85 teams	Cross-sectional	Project management simulation (building a scale model of a vehicle; 4–6)	Flow-scale [54] (quantitative, internal)	
Bakker et al. [34]	Sport	*N* = 398 youth and reserve soccer players (age: *M* = 17.5, *SD* = 2.2) of 15 professional soccer clubs	Cross-sectional	Playing soccer (soccer; 8–11)	Self-translated Dutch version of the Flow State Scale (FSS; [55]) (quantitative, internal)	
Culbertson et al. [29]	Education	*N* = 14 students (7 m, 7 f; age: *M* = 20.58, *SD* = 1.83) of one introductory university course	Longitudinal (four weeks with a total of 17 learning sessions of which 15 were afterwards rated for GF)	Learning (working on learning material; 14)	Self-constructed single item (quantitative, internal)	Understanding of and interest in material is related to GF; GF during knowledge acquisition is not related to quiz performance
Duff et al. [38]	Work				Proposed measure (unspecific): a variable to assess compensatory strategies (i.e. “team’s response to ‘flow disruptions’, p. 576) (quantitative, external)	
Gaggioli et al. [39]	Education				Proposed measure: Processual and structural features of collaboration: Social Network Analysis (SNA; [56, 57]) (quantitative, external)	
Gaggioli et al. [40]	Education	*N* = 30 undergraduate students (10 m, 20 f; age: *M* = 24.00, *SD* = 0.48) enrolled in a university course, divided into five groups	Longitudinal (twelve weeks; GF was measured at the beginning of week 2 and at the end of week 12)	Creative designing (designing a new technology-based psychological application; 5–7)	Adapted (items were framed in relation to the collective collaboration experience) Italian version of the FSS [55] (quantitative, internal)	Social support and performance feedback facilitates GF; GF is positively related to performance.
Gaggioli et al. [41]	Music	*N* = 75 amateur musicians (64 m, 11 f; age: *M* = 30.81, *SD* = 11.62) of 15 bands	Cross-sectional	Making music (unspecific; 3–7)	Italian version of the FSS [55] (quantitative, internal)	
Galimberti et al. [42]	Global				Proposed measures: (1) Intrapersonal level: measurement of quality of experience via adaption of FSS [55] and Networked Minds Measure of Social Presence [58]; (2) Interpersonal level: measurement of communicative interactions between participants based on interlocutory logic [59]; (3) Processual and structural features of collaboration: SNA [56, 57]; (4) Outcomes of collaboration: Creative Product Semantic Scale [60] (quantitative, internal/external)	
Gloor et al. [43]	Music	*N* = 8 jazz musicians of a professional band	Cross-sectional	Making music (giving a Jazz concert; 8)	Synchronized body movement within a group as measured by sociometric badges (quantitative, external)	Synchronization is a component of GF
Hart et al. [19]	Music	*N* = 6 musicians (4 m, 2 f; age: 20–22, *M* = 21) with experience of regularly playing music in a group	Cross-sectional	Making music (musical jamming; 6)	Semi-structured interviews, behavior observation of groups (qualitative, internal/external)	GF is a shared experience; GF and individual flow have the same characteristics (except clear goals and unambiguous feedback); development of empathy is a specific characteristic of GF
Heyne [30]	Work	*N* = 135 undergraduate students (70 m, 65 f; age: 18–25) divided into 45 teams	Experimental (between-subject design comparing low task complexity (basic task) vs. high task complexity (basic plus additional task))	Planning (providing humanitarian aid to a fictitious nation; 3)	Flow State Scale-2 (FSS-2; [28])^a^ (quantitative, internal)	
Kaye [44]	Gaming	*N* = 76 digital gamers (54 m, 22 f; age: 63.2% aged 18–25) with various levels of experience	Cross-sectional	Digital gaming (unspecific; unknown)	Adapted (items were framed in relation to group indicators of flow) and extended (five additional items to measure task-relevant knowledge of others, group cooperation, complementary participation, group feedback, group communication) version of the FSS-2 [28] (quantitative, internal)	Effective communication, knowledge of others´ skills and effective team work are determinants of GF
Kaye et al. [45]	Gaming	*N* = 17 digital gamers (16 m, 1 f; age: 18–40) divided into four semi-structured focus groups of four or five persons	Cross-sectional	Digital gaming (unspecific; unknown)	Qualitative analysis of open-ended focus group questions (qualitative, internal)	Collective competence, collaboration and task-relevant skills are antecedents of GF
Kaye et al. [46]	Gaming	*N* = 302 digital gamers (241 m, 61 f; age: 18–60, 62.67% aged 18–25)	Cross-sectional	Digital gaming (unspecific; unknown)	FSS [55]^b^ (quantitative, internal)	
Keeler et al. [27]	Music	*N* = 4 jazz vocalists (2 m, 2 f; age: > 18)	Quasi-experimental (2x2 within-subject design comparing standard (fixed composition) vs. improvised performance (composition with improvisation part) over time (pre vs. post))	Making music (vocal improvisation; 4)	FSS-2 [28]^a^ (quantitative, internal)	
Kiili et al. [47]	Physical Education	Study 1: *N* = 45 7th-9th graders Study 2: *N* = 60 7th-9th graders	Study 1: cross-sectional Study 2: cross-sectional	Study 1: Digital exertion gaming (playing “Tuck of War” or “Diamond Hunter”; 2–5) Study 2: Digital exertion gaming (playing “Speeding”; unknown)	Study 1: no GF measure Study 2: unknown measure of individual flow^b^ (unknown, unknown)	
MacDonald et al. [48]	Music	*N* = 45 music students (25 m, 20 f) split up in 15 groups	Longitudinal (unknown duration; unknown number of measurement time points)	Making music (creating a composition; 3)	Experience Sampling Method (ESM; [61])^a^ (quantitative, internal)	
Primus & Sonnenburg [49]	Work	*N* = 29 students (9 m, 20 f; age: *M* = 27.8, *SD* = 5.7) split up in six teams	Experimental (2x8mixed-measures design comparing two groups (task with creative warm-up activity first followed by task without warm-up-activity vs. task without warm-up activity first followed by task with warm-up activity) over time (4 measurement time points per task))	Design thinking (Lego Serious Play; unknown)	Self-constructed GF measure (quantitative, internal)	Group flow is positively related to individual flow; creative warm-up increases group flow
Ryu & Parsons [23]	Education	*N* = 45 college graduates (age: 20–28)	Experimental (between-subject design comparing instant collaboration vs. time-delayed collaboration vs. individual control condition)	Digital security guard training (digital mobile learning system; 2)	Six items adapted from Park, Parsons, & Ryu [62]^b^ (quantitative, internal)	
Salanova et al. [50]	Education	*N* = 250 university students (38 m, 212 f) in 52 small groups	Longitudinal study (three weeks with one session per week; two measurement time points (after the sessions of week 2 and week 3))	Event planning (developing and promoting a socio-cultural project; 5)	Self-constructed GF measure (quantitative, internal)	Collective efficacy is both an antecedent and consequence of collective flow
Sawyer [8]	Music	Experts from jazz, classical music and improvisational theater	Case descriptions, non-systematic literature review	Making music (unspecific; unknown)	No GF measure	Characteristics of group creativity are improvisation, collaboration and emergence of GF
Walker [14]	Study 1: Global Study 2: Gaming Study 3: Gaming	Study 1: *N* = 95 students (46 m, 49 f; age: *M* = 20.2); Study 2: *N* = 30 (14 m, 16 f; age: *M* = 19.8); Study 3: *N* = 48 (20 m, 28 f; age: *M* = 20.3)	Study 1: cross-sectional Study 2: experimental (within-subject-design comparing solitary (ball bouncing) vs. dyadic (volleying a ball between each other) paddleball) Study 3: experimental (between-subject design comparing high (dyadic ball passing) vs. low (interactive ball passing) interdependence)	Study 1: Global (unspecific; unknown) Study 2: Playing paddleball (volleying a ball; 2) Study 3: Playing pickleball (down-sized form of tennis; 2)	Study 1: open questions about examples of past flow experiences (qualitative, internal) Study 2: one item to measure the state participants felt most often (individual flow, boredom, apathy or anxiety; [61])^b^ (quantitative, internal) Study 3: one item to measure the state participants felt most often (individual flow, boredom, apathy or anxiety; [61])^b^ (quantitative, internal)	Study 1: participants gave fewer examples of solitary flow, with more examples of interactive GF than co-active GF
Zumeta et al. [52]	Sport and physical activity	*N* = 276 physically active students (196 m, 80 f); age: 19–30, *M* = 21, *SD* = 2.28)	Cross-sectional	Different sports and physical activities (various; various)	Shared Flow Scale (based on Dispositional Flow Scale; [52, 63]) (quantitative, internal)	GF mediates the relationship between group identification and collective efficacy
Zumeta et al. [53]	Music	*N* = 550 musicians (279 m, 271 f; age: 18–90, *M* = 42.75, *SD* = 13.98) of 52 drumming groups	Longitudinal (nine days; data collection four days before, at the end and four days after a drum festival)	Making music (playing drums; unknown)	Shared Flow Scale (based on Dispositional Flow Scale; [52, 63]) (quantitative; internal)	GF positively affects well-being, collective efficacy, fusion of identity with the group and social integration

GF = group flow; m = male; f = female; age is reported in years. Inconsistent sample characteristics appear due to different sample information between the publications. The column “main result” displays the main result of the publications in terms of group flow, not the main result of the publications per se.

^a^The results of this study were not included into the synthesis of empirical findings because the study measured individual flow which was aggregated to a GF value per group.

^b^The results of this study were not included into the synthesis of empirical findings because the study measured individual flow.

The measures used or proposed can be differentiated into different categories on two dimensions. On the first dimension (quality of data), publications differ in whether they used or suggested quantitative measures (20 publications) or qualitative (five publications) measures. On the second dimension (source of data), publications differ in whether they used measures that assessed group flow from an internal perspective of the group member(s) involved (e.g., self-report questionnaire; 20 publications) or from an external perspective (e.g., structured observation; six publications), with two publications using a combination of both an internal perspective measure and an external perspective measure. In one publication, the proposed measure was unclear. Measures assessing the internal perspective differ in whether they used an actual group flow measure (16 publications) or whether they measured individual flow experience in a group (i.e. every group member rates his/her own individual flow experience during a group task) and aggregated these individual flow values to a group flow value of the respective group (nine publications); additionally, one publication presenting three studies used both an actual group flow measure and an individual flow measure. Measures assessing individual flow experiences only (be it a self-report measure or a measure from an external perspective) and the related results will not be presented in this review because they do not fit the general group flow conceptualization (see also the Method section). Consequently, only the remaining 16 measures that actually assessed (a kind of) group flow and did not measure individual flow will be presented in the subsequent sections.

#### Quantitative measures

Eight publications used quantitative questionnaires or single-items to measure group flow from the internal perspective. Of these, five publications [40, 44, 52, 53] used or suggested questionnaires that were originally designed to measure individual flow but which were framed towards group flow. In more detail, two publications used [40] or suggested [42] an Italian version of the Flow State Scale (FSS; [55]), whereas Kaye [44] adapted and extended the Flow State Scale-2 (FSS-2; [28]) and Zumeta and colleagues [52, 53] adapted the Spanish version [63] of the Dispositional Flow Scale [55], asking the participants to relate the items of this questionnaire to their experience in the group. Three publications used a single-item (“Students in the class seemed to be ‘switched on’.”; [29], p. 33) or a questionnaire [49, 50] that the authors claimed were specifically developed to assess group flow. In more detail, Primus and Sonnenburg [49] used a self-constructed questionnaire based on seven items (e.g., items related to continuous communication among group members and the extent of everybody in the team participating equally) derived from Sawyers [26] group flow concept; Salanova and colleagues [50] used a self-constructed group flow questionnaire comprising three factors (group task absorption, group task enjoyment, group challenge and skills). Moreover, one of the aforementioned studies [42] made the additional suggestion to assess the quality of the group flow experience by adapting the Networked Minds Measures of Social Presence [58] and to assess the outcomes of group collaboration as a criterion of group flow with the use of the Product Semantic Scale [60].

Four publications used [43] or suggested [38, 39, 42] quantitative outcomes other than self-report questionnaires to measure group flow from an external perspective. In more detail, Gloor et al. [43] made use of sensors assessing body movement of every group member. The inter-individual comparison of group members’ activity level was taken as a measure of group flow, with inter-individually synchronous activity indicating group flow. Galimberti et al. [42] proposed two different measures of group flow from an external perspective for future investigations: firstly, on an interpersonal level, the communicative interactions between group members based on interlocutory logic [59]; secondly, on a processual and structural level of in-group collaboration, Social Network Analysis (SNA; [56, 57]). The use of SNA was also proposed by Gaggioli and colleagues [39]. Finally, Duff et al. [38] planned to measure group flow via team functioning with a variable that assesses compensatory strategies. Compensatory strategies can be defined as a group’s responses to group flow disruptions. The measure is intended to be applied after a flow disruption event, providing an observer the opportunity to rate a team’s actions in response to the given event.

#### Qualitative measures

Three publications [19, 31, 32] used structured observations of group flow. Based on different coding schemes, raters tried to identify whether group flow was present in a group or not (e.g., by identifying whether group members perform the same gesture simultaneously; [32]). Two publications [19, 45] used qualitative interviews to examine group flow, partly in combination with the aforementioned structured observations [19]. These interviews were analyzed with the use of Grounded Theory [19] and qualitative content analysis [45]. One publication [14] used a qualitative questionnaire asking the participants for open descriptions of group flow experiences.

### Empirical findings on group flow

The empirical findings included in this review do not comprise studies measuring group flow by assessing individual flow because this does not fit the general group flow conceptualization (see Introduction and Method section). An overview of the empirical findings of the remaining fourteen studies is displayed in Table 2. Empirical findings can be subdivided into antecedents, components (i.e. characteristics), consequences and correlates of group flow.

Studies identified competence-, (inter-)action and relationship-related antecedents of group flow. In terms of competence, a task-related collective warming-up [49], having task-relevant skills (as found in a cross-sectional, qualitative study in the context of digital gaming; [45]), knowing others’ skills (cross-sectional comparison study in the context of digital gaming; [44]), collective competence [45] and collective efficacy (longitudinal study in the context of work and education; [50]) were identified as antecedents. With regard to (inter-)action, collective collaboration [45], effective communication [44], decentralization within the group (cross-sectional observation study in the context of education; [32]), effective team work [44] and receiving performance feedback (longitudinal study in the context of music; [40]) were found to be influencing factors. Furthermore, in terms of relationship, trust within the group [32] and social support between group members [40] were identified as antecedents.

In terms of components of group flow, studies identified aspects of individual group members and aspects of the entire group being in group flow. With regard to individual aspects, Hart and Di Blasi (cross-sectional study in the context of music; [19]) found the same components in group flow as in individual flow except clear goals and unambiguous feedback. Regarding the entire group, studies found synchronized body movement (cross-sectional study in the context of music; [43]), interactional synchrony and mutual responsiveness (narrative, non-systematic literature review in the context of music; [8]).

In terms of consequences, longitudinal studies report several positive effects of group flow. Positive effects were found in terms of collective efficacy (in the contexts of work and music; [50, 52]), understanding of and interest in a task (in the context of education; [29]), well-being, fusion of identity with the group and social integration (in the context of music; [53]). Performance was not found to be a consequence of group flow (in the context of education; [29]).

Furthermore, particularly cross-sectional studies identified correlates of group flow. These studies identified positive relationships between group flow and individual flow [49], group flow and performance (in the contexts of education and work; [31, 40]), and group flow and development of empathy between group members (in the context of music; [19]). One study found group flow to mediate the relationship between group identification and collective efficacy (in the context of sport and physical activity; [15]). No relationship was found between group flow and learning (in the context of education; [31]). Finally, one separate study investigated the frequency of mention of group flow and individual flow examples, reporting that more group flow examples were given (across all possible contexts; [14]). Examples included “acting in a play on a night when everyone is on” (p. 5) for group flow or “writing a poem in the solitude of my family`s cabin” (p.5) for individual flow.

## Discussion

The overall purpose of the present paper was to review the existing literature on group flow, addressing four specific aims: firstly, to present an overview of recent definitions of group flow; secondly, to provide an overview of recent theoretical approaches to group flow; thirdly, to review recent measures to assess group flow; and fourthly, to review empirical findings on antecedent conditions, components, and consequences of group flow. Twenty-six English language, peer-reviewed publications addressing group flow were identified. These publications utilized non-systematic, narrative literature reviews and theoretical considerations as well as cross-sectional and longitudinal studies. In general, the definitions, theoretical approaches and measures of group flow stated within the included publications were highly heterogeneous. Although the concept of group flow initially emerged from the concept of individual flow [7] and, thus, from the field of psychology, it has been examined from the perspectives of different fields (e.g., psychology, sociology) and interdisciplinary fields (e.g., organizational behavior). The empirical publications identified in this scoping review give an initial indication of the antecedents, components, consequences, and correlates of group flow. The results of the scoping review will be discussed in the following section with respect to the four aims, taking into account the limitations of the reviewed publications.

### Definitions of group flow

The definitions of group flow stated in the included publications were very heterogeneous in their *content*. Firstly, group flow is defined from an individual perspective (i.e. how an individual experiences group flow) and a collective perspective (i.e. features of a group as a whole being in flow), with some definitions comprising both perspectives and others only comprising the collective perspective. At first sight, this reflects a different conceptualization of group flow among researchers. However, this may reflect only a different emphasis when looking at group flow.

Secondly, there was a difference in the *content of the collective aspects* (i.e. shared state, group interaction, social constellation, and group performance) mentioned in the definitions. Whereas the shared state more generally represents that group flow is something group members collectively share, group interaction, social constellation, and group performance appear to be collective aspects that contribute to or make up the constitution of this shared state. Many definitions focused only on the shared state and on performance, but not on the social constellation or the interaction. Other definitions did not focus on performance, but on the social constellation or the interaction. Again, at first sight, this may also reflect a different understanding of group flow between researchers with regard to the specific content of the collective aspects and group flow in general. However, these variations might only be a consequence of the fact that group flow has been studied across a broad range of contexts (see Table 2). The content of different contexts might set different emphases on the construct of group flow. For instance, performance–as a rigorous output–is more relevant to some domains (e.g., work) than to others (e.g., leisure time activities), which, in turn, sets a different focus in the definitions of group flow. Therefore, the differing collective aspects mentioned in the definitions should be seen as separate pieces of a single puzzle that come together to form the whole. For instance, if one definition focuses on the shared state only (for example, Gaggioli et al. [41] defined group flow only by “a ‘collective state of mind’” (p. 2)), this does not contradict definitions that address (also) performance and interaction (for example, Keeler et al. [27] defined group flow by an “optimal performance, but also optimal interaction with others” (p. 3)).

Moreover, definitions of group flow were heterogeneous with regard to their *formal characteristics*. Firstly, the wording of a number of definitions included references to the state of group flow as well as antecedents or consequences of group flow (e.g., group flow *requires* the mere presence of others; [42]). Although antecedents and consequences might not reflect the state of group flow per se, the inclusion of such process-related variables allows for a description of criteria of group flow. Secondly, some definitions were imprecise and hard to operationalize (e.g., transcendence of ego; [52]) whereas others were not.

The heterogeneity of definitions leads to a variety of problems. Above all, it prevents clear communication between researchers. Furthermore, it impedes theory building, as theory building requires a clear definition of an endogenous construct of interest [64], and it may lead to different and even inconsistent operationalizations of group flow.

As an overall consequence, there is a need for a comprehensive *integrative definition* of group flow. Such an integrative definition should meet two criteria. Firstly, it should take into account previous approaches to define group flow, existing theoretical approaches to describe and explain flow, and empirical findings on group flow as identified in this review. Secondly, such an integrative definition should have a description of the *state* of group flow at its core (i.e. the state-related components of group flow), completed by antecedents and consequences of group flow (i.e. process-related components of group flow). As mentioned before, although antecedents and consequences of group flow might not reflect the state of group flow per se, the inclusion of such process-related variables allows for a description of criteria of group flow.

A systematic decomposition of all available approaches to define group flow, existing theoretical approaches to group flow, and previous empirical findings on group flow allows the identification of the elements that appear to be an appropriate part of an integrative definition. Above all, group flow is a state that group members share (e.g., see [50] for previous definitional approaches; see [19] for previous empirical findings). Thereupon, *components* of group flow which systematically and consistently complement each other are positive, fluent interactions within the group (e.g., see [27] for previous definitional approaches; see [26] for a theoretical description; see [43] for previous empirical findings), a high level of competence of the group in order to fulfill the group task (e.g., see [14] for previous definitional approaches; see [47] for a theoretical description; see [45] for previous empirical findings), and a collective state of mind (e.g., see [39] for previous definitional approaches; see [50] for a theoretical description; see [19] for previous empirical findings). The occurrence of these components as a reflection of a specific form of balance in the group is made possible through positive relationships between group members as a central *antecedent* (e.g., see [46] for previous definitional approaches; see [65] for a theoretical explanation; see [52] for previous empirical findings). Finally, central *consequences* of group flow are performance (e.g., see [43] for previous definitional approaches; see [31] for previous empirical findings) and creativity (e.g., see [42] for previous definitional approaches; see [8] for previous empirical findings) which, taken together with the components, make group flow a positive experience (e.g., see [40] for previous definitional approaches; see [19] for previous empirical findings).

Thus, considering the criteria for an integrative definition listed above, and the decomposition of the existing approaches and findings, we suggest the synthesis of the existing approaches and findings in the following integrative working definition of group flow: *Group flow is a shared state of balance within a group as represented by (a) fluent*, *positive interactions within the group*, *(b) a high collective competence of the group and (c) a collective state of mind of the group by means of positive relationships between group members*, *often resulting in optimal collective performance and creativity*, *and making group flow a positive collective experience*. As a corollary, group flow is a state that can be *observed* on a group level, either from the internal perspective of a group member involved or from an external perspective (e.g., by observing fluent interactions within a group), and is *experienced* and *rated* subjectively by the group members on the individual level (e.g., having the positive experience).

### Theoretical approaches to group flow

Less than half of the reviewed publications presented, used or elaborated a specific theoretical framework. The lack of a theoretical framework was particularly evident in empirical investigations, which reflects the general lack of theoretical underpinnings in some fields of empirical research (e.g., [66]). This shortcoming restricts the interpretability of study results as not using a theory diminishes the explanatory and predictive value of empirical findings (for an overview, see [67]).

The existing theoretical approaches to group flow differ in the *focus* of what they describe and explain, and in their *context-specificity*. The *focus of description and explanation* relates to a differing consideration of two dimensions, that is, (a) whether and how the theoretical approaches describe the *state* of group flow and (b) whether and how the theoretical approaches describe and explain the *processes* around group flow (e.g., causal mechanisms for its occurrence). For instance, the Group Flow concept by Sawyer [26] both (a) describes the group flow state (e.g., being in interactional synchrony) and (b) describes and explains the underlying processes (e.g., parallel processing between group members).

Regarding the *state* of group flow, all theoretical approaches consistently describe *balance* within the group as a major characteristic. Although several different words for balance are used (e.g., balance, similarity, synchrony), these theoretical approaches only significantly differ with regard to the object of the balance that is said to be present in a group during group flow: For example, the channel model of group flow by Kiili and colleagues [47] describes a balance in terms of the group competence (balance between the group’s overall skills and the challenges), Salanova and colleagues [50] describe a balance in terms of the group’s state of mind (balance between challenge and group efficacy beliefs), and the Group Flow concept by Sawyer [26] additionally describes a balance in the group’s behavior (interactional synchrony).

The balance within the group as a major characteristic of group flow provides a primary explanation as to why group flow differs from (an aggregation of) individual flow experiences. During individual flow, there is, for example, a balance between an individual’s task-related challenges (be it a task during a social situation or a task in a solitary situation) and the individual’s skills. In contrast, during group flow, the balance additionally includes all group members. This balance inevitably makes group flow a specific experience: It occurs between group members as they have something in common that is perceived by everyone (e.g., group resources, group efficacy beliefs). Thus, group flow is not only related to one’s individual action and experience and is, therefore, qualitatively different from individual flow.

In terms of the *processes* around group flow, there was a high heterogeneity between theoretical approaches. These theoretical approaches implicitly describe proximal and distal processes. Proximal processes describe and explain how a group enters (i.e. how it transitions from imbalance to balance), loses (i.e. how it transitions from balance to imbalance), or changes (i.e. how it transitions from balance A to balance B) the state of group flow. For instance, the Group Flow concept by Sawyer [26], assumes parallel processing (i.e. simultaneously concentrating on and responding to each other) between group members to be important for reaching and maintaining group flow. Distal processes describe and explain how general preceding factors facilitate or impede the occurrence of group flow and the consequences of group flow. For example, the Networked Flow Model [39] assumes that persistence across group members (e.g., recognizing other’s intentions) facilitates the occurrence of group flow.

The theoretical assumptions of the processes around group flow provide further explanations as to why group flow differs from individual flow experiences. In terms of individual flow, for example, processes influencing the occurrence of individual flow are often perceived as being controllable by the respective individual (for an overview, see [2]). In contrast, with regard to group flow, the group context inevitably makes group flow less controllable by introducing many additional variables and specific processes [14].

Taken together, the theoretical description and explanation of the group flow state and its integral processes also allow distinctions to be made between group flow and allegedly similar constructs. Such similar constructs are often potential causes or consequences (e.g., group synergy) of group flow or social processes (e.g., social facilitation) related to an individual’s task that have to be distinguished from processes concerning group tasks. For example, group synergy seems to be a potential consequence of group flow. Just like creativity, which is typically assumed to be a consequence of group flow (see [8, 15, 26]), group synergy (i.e. the event that a group generates more creative thoughts than the sum of the individuals within the respective group; [68]) reflects a group gain [69] that may result from group flow (e.g., due to a balance in common goals). In contrast, social facilitation (i.e. the tendency of individuals to perform better during the presence of others under certain circumstances; [70]), for instance, is a social process related to an individual’s task accomplishment in a social situation (e.g., with an audience being present) and not to the accomplishment of a group task. Thus, social facilitation can be associated with individual flow in a social situation but not with group flow.

Besides differing in focus of description and explanation, the existing theoretical approaches also differ in their *context-specificity*. Whereas some theoretical approaches relate to a specific context, others are unspecific and can be applied to any context. For instance, the multi-level model of flow in sociotechnical systems [38] was specifically designed to be applied in the work context; in contrast, Networked Flow Model [39, 41, 42] is context-unspecific.

Taking all the existing theoretical approaches together, a context-unspecific theoretical description of the group flow state and a description and explanation of the processes surrounding group flow are important for a better understanding of group flow. For this reason, there is a need for the development of a comprehensive theoretical model that brings together the multitude of existing theoretical approaches that–if taken separately–can only describe and explain specific aspects of group flow, partly also only in specific contexts. Such a theoretical model should first describe the state of group flow before including processes that describe and explain the occurrence, change, and consequences of the state. To date, the existing theoretical approaches do not systematically link aspects of state and processes. Social psychology offers strong, well-established theories to potentially describe and explain this link (e.g., Social Identity Theory, [71]; Optimal Distinctiveness Theory, [72]; Balance Theory, [65]). For instance, Heider’s balance theory [65] offers a suitable framework by describing and explaining how balance (as a major characteristic of group flow) in social systems occurs due to a positive relationship between the individuals involved (as a major antecedent of group flow). Finally, a comprehensive theoretical model of group flow should be empirically tested because falsifiability and predictive power are primary criteria of well-elaborated scientific theories. Until now, few publications have empirically tested (e.g., [50]) their theoretical group flow approach.

### Measures of group flow

The existing measures and the measures proposed can be distinguished on two dimensions. First, instruments differ in whether their underlying approach is quantitative or qualitative in nature (quality of data). Second, instruments differ in whether they assess group flow from an internal perspective or from an external perspective (source of data). Of the existing instruments, some were specifically designed for group flow, whereas others were adapted for group flow; some studies used validated instruments, but the overwhelming majority used non-validated instruments or questionable approaches (e.g., single item measures).

In general, the differentiation of group flow measures on the two dimensions allows for the application of methodological triangulation. Triangulation (i.e. using different methods to investigate one phenomenon) can result in a new picture or a different construction of an object [73]. For group flow, triangulation is particularly useful because it allows for assessing group flow from an internal and an external perspective, both of which forming part of the corollary of our integrative definition. This is in line with previous literature explicitly suggesting the process of triangulation for group flow (cf. [42]).

In addition to the existing approaches, a future consideration of a time dimension seems to be reasonable. To date, most of the existing measures (in particular, the self-report instruments) retrospectively assess group flow. In contrast, instruments that capture momentary group flow (i.e. measuring group flow during an activity) are scarce. Those instruments would allow for an examination of temporal stability and dynamics of group flow.

In conclusion, the state of group flow measures in general and the specific demand for a time dimension defines tasks for the (further) development of instruments assessing group flow. Above all, all existing and future measures should be appropriately validated and applied. Each instrument requires validation, which was not performed for every measure used in the reviewed studies. Based on this general position, the (further) development of instruments should at best result in a complete set of instruments. This means, that, at best, there should be instruments for each combination of the three dimensions (i.e. quality of data, source of data, time). In order to provide such a variety, it is particularly necessary to advance measures with regard to the source of data and the time dimension. With regard to the source of data, the further development of the assessment of the internal perspective could begin (1) with qualitative interviews regarding antecedents, components and consequences of group flow to generate items and (2) by making use of previous subjective balance measures (see [74]) as balance was identified as a central characteristic of group flow. A subsequent systematic validation of such a measure should include examinations of the construct validity, for instance, through discriminant and convergent validity regarding individual flow and group cohesion [75]. In terms of the external perspective, future measures should be able to measure group interactions (e.g., with the use of interaction analysis; [76]) and consequences of group action as criteria of group flow (e.g., objective group products; [42]). This can be facilitated by interdisciplinary collaboration in the study of small groups (see [77, 78]) making use of, for example, social and computer science at the same time [79]. Also, studies should think of using objective physiological measures (e.g., heart rate, cortisol, brain activity) which were shown to be associated with individual flow [80, 81, 82]. The resulting group flow measures could then also be used for cross-validation among themselves (e.g., correlating a quantitative self-report measure for the internal perspective with an observation measure for the external perspective).

With regard to the time dimension, an entirely new development of assessments of momentary group flow is necessary. For individual flow, classical research has used the Experience Sampling Method [61] and current research has used software applications on mobile devices [83]. Similar procedures should also be applied for group flow.

### Empirical findings on group flow

Studies show that group flow can occur in different tasks and contexts and is characterized by specific components, several antecedents, and outcomes. In essence, studies found individual aspects (e.g., loss of self-consciousness; [19]) and collective aspects of the group (e.g., synchronization; [43]) to be components of group flow. Moreover, studies identified aspects of competence (e.g., knowing others’ skills; [45]), interaction (e.g., effective communication; [44]), and of positive relationships (e.g., trust within the group; [32]) to be antecedents of group flow and several positive individual (e.g., well-being; [53]) and group-related (e.g., collective efficacy; [50]) outcomes.

The findings reveal that some aspects of group flow seem to be similar to individual flow, but that there are also aspects that are unique to group flow. For example, Hart and Di Blasi [19] found the same characteristics in group flow as found in individual flow, except for clear goals and unambiguous feedback; in contrast, Sawyer [8], for instance, found interactional synchrony to be a unique component of group flow. An analogical situation occurs for antecedents, with task-relevant competence (e.g., [45]) reflecting similarities to individual flow while aspects like trust within a group [32] incorporating the uniqueness of group flow. Taken together, this confirms previous assumptions (e.g., [9, 14, 19]) that group flow has qualitative differences to individual flow and surpasses a simple feeling of individual flow in a group setting.

There are methodological and theoretical approaches to explain why some aspects might be similar to individual flow whereas others are unique to group flow. From a methodological point of view, recent group flow measures function as an explanation: As some, but not all measures were based on individual flow questionnaires asking the participants to relate the items of the questionnaire to their experience in the group, this can explain why some studies trivially found similarities whilst others did not (see also [84]). From a theoretical point of view, above all, it is important to take into account the global statement that individuals act, think, and feel differently during group situations in comparison to solitary situations [14], which logically introduces unique aspects of group flow. However, the specific theoretical underpinnings are not yet established [44]. Future studies should, for example, test individual and group-related influencing factors. In terms of individual factors, for instance, the affiliation motive was found to be positively related to individual flow during social situations [85], perhaps because persons with a strong affiliation motive have developed better social skills and have, thus, the opportunity to act adequately in social situations [13]. This could also be important for group flow. In terms of group-related factors, studies could take into account contagion processes between group members (e.g., [72, 86, 87]).

There are some methodological limitations to the interpretability and the generalizability of the reviewed studies. Besides the aforementioned measurement issues, these limitations are to be found in the contexts of investigation, the study designs, and the procedures of data analysis. As the studies included different contexts (work, education, music, sport, digital gaming), specific results cannot be generalized because group tasks in different context have many different, specific facets (e.g., playing music in an orchestra vs. collaborative learning during education). In terms of study designs, the majority of studies were cross-sectional in nature, reducing their ability to explain causal mechanisms. Finally, another limitation relates to mixed levels of data analysis as outlined by Gully, Devine, and Whitney [88]. Some studies performed measurements at the individual level (i.e. they took into account the subjective perspective of the group members), whereas others measured at the group level (i.e. from an external perspective). Besides the theoretical difference between these two approaches, a comparison of the results measured on different levels can lead to under- or overestimations when sample-size weights are not taken into account.

Irrespective of the methodological limitations, the current state of research reveals a range of research gaps with regard to group flow in any case. These gaps are obviously present for the identification of components, antecedents and consequences of group flow, but they are also present for the identification of potential moderators. The identification of potential moderators of the relationship between antecedents and group flow, and of the relationship between group flow and its consequences has been entirely neglected to date. Potential moderators could be found in the characteristics of a group but also in the group task. With regard to the group, factors such as role clarity (e.g., influencing the formation of balance within the group) or group size (e.g., influencing the communication style between group members) could be moderators. In terms of the task, the social task condition could be a moderator (e.g., a proactive-reactive task probably influences interpersonal behavior in another way than a coactive task). Thus, taken together, both the research gaps and the methodological limitations derive the need for a systematic research program.

### General conclusion

Overall, the aforementioned potential benefits of this scoping review were only partly fulfilled according to the current state of research. On the one hand, our review paves the way for a common understanding of and future research on group flow by revealing definitional and theoretical problems, research gaps and methodological limitations. On the other hand, however, the review does not allow for a practical application of a group flow concept just yet.

In terms of future research, this review highlights the need for a systematic research program. First, this research program consists of conceptual work that needs to be carried out in a sequential order ((a) to (d)), followed by empirical investigations ((d) to (f)) that can, at least in part, take place simultaneously. More specifically, the research program should comprise (a) finding a consensus for the integrative definition of group flow (e.g., by disseminating the integrative definition and by seeking feedback on it from other experts in the field) and (b) developing a comprehensive theoretical model of group flow first (e.g., based on well-established theories of social psychology as conglomerating elements between several existing theoretical orientations). Subsequently, (c) the aforementioned comprehensive development of consistent and theory-based measures of group flow should follow taking into account the three dimensions of quality of data, source of data and time. Existing measures will then facilitate (d) the realization of different kinds of experimental studies on antecedents and consequences of group flow (e.g., laboratory studies testing the influence of emotional contagion on the state of group flow). In so doing, researchers should more consistently avoid building and relying on studies that do not explicitly focus on group flow or do not measure group flow in the narrower sense (although they claim to measure it) than was done in the past (e.g., [27, 89]). Moreover, experimental studies will allow for (e) an identification of moderators (e.g., studies could systematically test Steiner’s taxonomy [90] of social task structures as an independent variable). Also, (f) theory-based interventions should be developed and evaluated (e.g., interventions similar to individual flow interventions; see [2]). Finally, in the long-term, with the fulfillment of these steps the completion of a meta-analysis will be possible, as this requires a sufficient number of studies that are similar enough to each other [91].

Regarding practical implications, our findings provide some insights into the potentially beneficial effects of group flow. It is conceivable that group flow experiences have positive consequences for the group as well as for the individuals that constitute the respective group and the society the individuals and the group belong to. Literature in the field of individual flow [5] presages that group flow may also occur in any other imaginable context of our everyday life in which a group and a group task are present. This, in turn, highlights the need to take into account the potential benefits (e.g., well-being) of group flow (as a positive social experience) for society in general given the many societal challenges we currently face (e.g., inclusive society, social injustice) but also for specific groups (e.g., work group, music group, sport team). To date, the existing literature particularly suggests that group flow can be facilitated or enhanced by an increase in the level of competence of a group, by an improvement of interaction and by an improvement of social relationships. Therefore, future studies should not only investigate the conditions under which group flow occurs, but also how these conditions can be created on different levels, that is, by group members (e.g., workers, musicians, athletes), group leaders (e.g., by leaders of a work group, maestros of a music group, coaches of a sports team), and respective superordinate organizations (e.g., companies, clubs) or societal institutions (e.g., schools). One approach to examine how these conditions can be created could be to build upon recent work on group reflexivity [92, 93].
